# Population and Climate Change: Consensus and Dissensus among Demographers

**DOI:** 10.1007/s10680-021-09580-6

**Published:** 2021-03-25

**Authors:** Hendrik P. van Dalen, Kène Henkens

**Affiliations:** 1 Netherlands Interdisciplinary Demographic Institute (NIDI-KNAW), P.O. Box 11650, NL-2502 AR The Hague, The Netherlands; 2 Tilburg School of Economics and Management (TISEM), Tilburg University, P.O. Box 90153, NL-5000 LE Tilburg, The Netherlands; 3 University of Groningen, University Medical Center Groningen (UMCG), P.O. Box 72, NL-9700 AB Groningen, The Netherlands; 4 Department of Sociology, University of Amsterdam, Nieuwe Achtergracht 166, NL-1018 WV Amsterdam, The Netherlands

**Keywords:** Climate change, Population policy, Population decline, Fertility, Demographers, Family planning

## Abstract

What role does population play in thinking about the problem of climate change and some of its solutions? In a survey conducted between February and April 2020, we asked European demographers to state their views on the relationship between climate change and population developments, and asked them to rate their concern about climate change and other socio-demographic issues. We found that climate change is at the top of the list of demographers’ concerns, but that their sense of urgency with respect to taking action to redress global warming is not matched by their belief that population policy can make a crucial difference in reducing CO_2_ emissions: demographers are highly divided on the question whether the global population size should be reduced to lower CO_2_ emissions, as well as on the question whether family planning is an effective policy instrument.

## Introduction

Climate change is a topic of great concern, and scholars from different disciplines try to provide answers on how to reduce global emissions and adapt society to the consequences of global warming. Some prominent demographers have engaged in the debate over the years by giving their view of how population and global warming are intertwined (Bongaarts 1992; Bongaarts and O'Neill 2018; Dyson 2005; Lutz 2017; O'Neill et al. 2005). They argue that curtailing world population growth is crucial for combatting the root causes of climate change. In particular, Bongaarts and O’Neill (2018) stress the importance of voluntary family planning as a climate policy lever.^1^ This preference for population policies seems to be even stronger among scientists from outside the field of demography (Crist et al. 2017; Gerlagh et al. 2018; Guillebaud 2016). Biologists, ecologists, and economists all seem to have no qualms about suggesting how population policies could play a major role in limiting world population growth. For instance, close to 14,000 scientists worldwide offered ‘a warning to humanity’ about our failure to make sufficient progress in solving the predicted environmental challenges, and in their pamphlet, one of their proposals towards sustainability involves ‘reducing fertility rates’ (Ripple et al. 2017). However, these early suggestions were apparently not sufficiently persuasive, as they ended up getting left out in the influential reviews and assessment by the Intergovernmental Panel on Climate Change (IPCC) (2014). This panel of climate change experts acknowledges population growth as one of the key drivers, but when they write about corrective policy measures they leave out the option of population policies.

With increasing pressure on governments to implement measures to reduce global CO_2_ emissions, the opinions of demographers may become more relevant in addressing future challenges. Opinions of demographers can be important because these experts are likely to inform policy makers directly or indirectly, for example by cultivating a long-term perspective on population developments. Furthermore, demography is the science that is in principle in the best position to provide reliable answers about the potential role of population policy, although it must be admitted that the bulk of demographic research rarely focuses on the interaction between population and climate change. This study is the first to provide results from a survey among demographers on how important an issue they consider climate change to be, and the role that population policies can play in reducing global warming. The results of this study show to what extent demographers have a shared view on the population-climate change nexus and whether this view is in line with the concerns formulated by the earlier mentioned prominent demographic scholars.

## Method and Data

In the three-month period of February-April 2020, the Netherlands Interdisciplinary Demographic Institute held an online survey among the members of the European Association for Population Studies (EAPS). The questionnaire was relatively short and the average time that it took people to fill in the questionnaire was ten minutes. Some of the questions were based on an earlier survey for demographers conducted by Van Dalen and Henkens (2012), but most questions in the current survey were geared towards understanding the demographic challenges (fertility, population decline, migration and climate change) that Europe faces.

To conform to European privacy regulations, the survey was distributed through the office of EAPS. The response rate was 30% (220 respondents of total 737 members that were registered as of 1 May 2020), which is perhaps low compared to general population-based surveys or surveys that use incentives, but it is in line with other expert surveys (Klein and Stern 2005; May et al. 2018; Van Dalen, 2019; Van Dalen and Henkens 2012).^2^ Respondents did not answer every question—they were allowed to skip a question if they wanted. As a result, the sample sizes vary slightly for the individual items.

The large majority of the respondents hold senior positions within their organizations: 29% of them are full professors and 43% of them are associate professors or senior researchers. Most of the demographers report that their knowledge of key demographic developments is medium to high (see Table 1). Knowledge of labour market issues is a relatively weaker point among respondents.

**Table 1:** Self-reported level of knowledge on demographic topics (percentages), 2020

Level of knowledge	Fertility, reproductive health	Health, mortality, life expectancy	Migration	Family relations	Labour market
	Percentages				
Low	9	13	13	14	22
Medium	37	47	46	44	57
High	55	41	41	42	22
Total	100	100	100	100	100

*N* = 189. Due to rounding errors, the sum of percentages may not always add up to 100

The gender composition of the sample is evenly distributed (49% men, 51% women), and the age distribution of respondents is: 44 years or younger (51%), 45–64 years (37%) and 65 years and older (12%), and naturally most of the respondents live in Europe (only 7% live outside Europe). To assess the representativeness of the sample, we compared the age and gender distribution with the membership records available from EAPS. This comparison is complicated, however, by the large numbers of missing values in these records.^3^ We find that the current sample is slightly younger: 36% of the members (according to EAPS membership records) are 44 years or younger, and 16% are 65 years or older. The percentage of women (51%) is more or less in line with EAPS membership records (56% are female).

## Concerns of Demographers

To ascertain whether climate change is a big concern among demographers, we asked respondents to express their worries or concerns about a series of societal issues, some of which are clearly demographic (e.g. low fertility, population decline), whereas others are of broader concern, like income inequality and climate change. Respondents were asked to answer the question ‘How worried are you about the development of the following issues in your country (of residence) for the next 20 years?’ with answer categories varying from ‘not at all worried’ to ‘extremely worried’. Table 2 gives an overview of the answers and ranks them by the percentage of respondents who reported being ‘extremely worried’.

**Table 2:** Worries of demographers about the future state of their country of residence, ranked by proportion of ‘extremely worried’, 2020

	Not at all worried	Not so worried	Somewhat worried	Very worried	Extremely worried	Don’t know	Total
	Percentages						
1. Climate change	2	7	22	34	35	1	100
2. Income inequality	2	5	28	38	27	0	100
3. Poverty	2	15	26	35	22	0	100
4. Discrimination^a^	1	19	30	31	20	0	100
5. Integration of immigrants	2	15	30	34	19	1	100
6. Gender inequality	3	25	31	31	10	0	100
7. Obesity	8	21	39	23	9	1	100
8. Low fertility	19	33	28	15	6	0	100
9. Large-scale immigration	12	34	37	11	6	0	100
10. Population decline	38	30	16	10	6	1	100

The question upon which these responses are based was stated as follows: ‘How worried are you about the development of the following issues in your country (of residence) for the next 20 years?’ with answer categories: (1) not at all worried; (2) not so worried; (3) somewhat worried; (4) very worried; (5) extremely worried; (6) don’t know. N varies for the individual items between 216 and 220. Due to rounding errors, the sum of percentages may not always add up to 100

(a) The questionnaire specified it thus: Discrimination based on age, gender, religion or ethnicity

A number of observations can be made. First of all, climate change is at the top of demographers’ minds, as evidenced by more than two-thirds of the respondents being very to extremely worried about this issue. Besides the issue of climate change, income inequality and poverty also are among the top-rated concerns among demographers. Second, typical demographic issues that figure prominently in top demography journals—like low fertility, population decline and immigration—are at the bottom of their list of worries.

To see whether the worries described in Table 2 differ according to relevant subgroups (e.g. geographical location), we have carried out an ordered logit analysis (Table 3) with (1) country context, (2) political orientation and (3) gender as independent variables. We used as control variables age and the rank within universities/institutes as a proxy for knowledge level (not in Table 3).

**Table 3:** Explaining worries of demographers

	Country of residence, by region (Northern Europe = 0)			Political orientation (× 10^–2^)	Gender (male = 0)	Pseudo-R^2^
	Southern Europe	Eastern Europe	Outside Europe			
Climate change	−0.34	0.27	1.11	−2.88***	−0.17	0.05
	(0.36)	(0.52)	(0.65)	(0.99)	(0.30)	
Income inequality	0.18	0.54	1.75**	−5.43***	−0.14	0.09
	(0.38)	(0.50)	(0.70)	(1.04)	(0.29)	
Poverty	0.85**	0.48	1.32**	−3.81***	0.09	0.06
	(0.37)	(0.49)	(0.61)	(0.95)	(0.32)	
Discrimination	−0.34	0.27	1.11	−2.88***	0.29	0.05
	(0.36)	(0.52)	(0.65)	(0.99)	(0.29)	
Integration of immigrants	1.09***	−0.79	1.35**	−0.15	0.24	0.06
	(0.36)	(0.52)	(0.66)	(0.93)	(0.24)	
Gender inequality	0.47	0.25	1.11*	−2.31**	0.62	0.08
	(0.36)	(0.51)	(0.67)	(0.97)	(0.29)	
Obesity	−0.11	0.01	0.85	0.12	−0.01	0.02
	(0.37)	(0.51)	(0.69)	(0.92)	(0.29)	
Low fertility	2.90***	0.53	0.09	3.05***	0.06	0.13
	(0.42)	(0.52)	(0.62)	(0.95)	(0.21)	
Large-scale immigration	0.15	−0.25	0.83	2.64***	−0.00	0.05
	(0.37)	(0.53)	(0.61)	(0.95)	(0.29)	
Population decline	2.13***	1.07**	0.31	1.97**	0.29	0.12
	(0.40)	(0.53)	(0.63)	(1.00)	(0.31)	

(a)These ordered logit estimates have also been controlled for age and professional rank within university/institute of respondents. The ‘don’t know’-category has been left out of the analyses. ***p < 0.01; **p < 0.05; and *p < 0.10. N varies between 186 and 188

The country context is relevant because the survey questions with respect to respondents’ concerns refer explicitly to their country of residence. It may very well be the case that the uniformity displayed in Table 1 is not present when one takes a look at the concerns of demographers from specific countries. The sample is unfortunately too small to show the effects per country, but one can show the effects for a number of regions within Europe. We distinguish four regions to which the respondents belong: Northern Europe, Southern Europe, Eastern Europe and outside Europe. We also asked respondents to place their political orientation on a sliding scale (100 points) from left to right. Political orientation is important because one’s stance on the issues, and certainly perceptions about climate change, are expected to be influenced by one’s political views (Carlton et al. 2015). One would also expect that population decline and low fertility are issues that worry respondents on the political right more than those on the left (cf. Van Dalen and Henkens (2020), considering that those leaning towards the right are more conservative and desire to maintain the status quo than those who lean towards the left. The measured political orientation is, however, quite skewed: 75% of the respondents see themselves as left-leaning (0–39 pts), 21% in the middle (40–59 pts) and 4% as right-leaning (60–100 pts).^4^ This distribution is in line with a European study of the political orientation of professors by Van de Werfhorst (2020) and has also been documented in American academic settings, where social scientists are known for placing themselves on the left of the political spectrum (Gross and Fosse 2012). Finally, we expect that concerns about gender inequality and discrimination (based on gender) may be affected by the gender of respondents. The descriptive statistics of these variables are given in Table 6 (see appendix).

Three estimation results from Table 3 merit attention. First of all, political orientation is important in understanding the diversity of concerns among demographers. For example, left-leaning demographers are much more persuaded than right-leaning demographers that climate change is a threat. Those on the political left also worry more about issues such as income inequality, poverty and gender inequality, whereas those on the political right are more concerned about demographic issues (low fertility, large-scale immigration and population decline). The political influence is, however, not that large that they overturn the ranking of issues with the current sample of demographers.^5^

Second, worries about societal and demographic developments also differ by the respondents’ region of residence. Compared to Northern Europe, demographers residing in Southern Europe are considerably more worried about population decline, low fertility, the integration of immigrants and poverty. The issue of population decline is also a bigger worry to demographers in Eastern Europe than it is among Northern European demographers. And a final observation is that the gender of a demographer does not matter when it comes to worries about gender inequality or discrimination.

## Population and Climate Change

The finding that demographers put climate change as the most worrisome development for the next 20 years raises several questions. First there is the question on how demographers view the connection between population and climate change, and second, what contribution can population policies make in decreasing the risks of climate change? The most visible demographer at this point in time is John Bongaarts, who makes a strong case for putting demography on the agenda of climate change discussions, and who argues that voluntary family planning is a suitable policy instrument to decrease the global population. In persuading policy makers and climate experts—the IPCC in particular—Bongaarts and O'Neill (2018) wrote an article in *Science* about the possible reasons why population policies are not considered. They formulate a number of popular misperceptions held by the climate policy makers and advisors about the role of population and the effectiveness of population policies in particular, namely:

(1) *Population growth is no longer a problem*. This misperception is according to Bongaarts and O’Neill in large part based on ‘the belief that fertility declines already under way in Asia and Latin America would soon occur in Africa; [as well as] the expectation that high AIDS mortality would halt population growth in Sub-Saharan Africa” (p. 651). Furthermore, earlier predictions made during the sixties and seventies about the negative consequences of high population growth did not materialize.

(2) *Population does not matter much for climate*. The misperception is based on the widespread belief that ‘past and current emissions have been attributed primarily to economic growth powered by fossil fuels in the currently high-income countries. [..] Although slower future population growth would not be the most important means of reducing future emissions, it could reduce global emissions by 40% or more in the long term’ (p. 651 and see also O'Neill et al. (2012)).

(3) *Population policies are not effective*. This perception is perhaps the most difficult to assess, but it can nevertheless hamper the implementation of policies. According to Bongaarts and O’Neill, ‘Family planning programs to assist women in achieving their reproductive goals […] have been successful in a number of countries, but further investments are still needed. Each year 85 million unintended pregnancies result in 32 million unplanned births worldwide. Population growth can be reduced substantially by avoiding these unplanned pregnancies.’ (p. 651).

(4) *Population policy is too controversial to succeed*. Family planning issues have always generated aversion or criticism based on religious principles or because in the past it has been associated with coercive measures, and when it comes to climate change specifically, Bongaarts and O’Neill note another stumbling block: ‘Many in the climate change community believe that entering into a population policy discussion thus blames the poor countries for problems created by the rich countries.’ (p. 652).

To test the extent to which demographers consider these issues raised by Bongaarts and O’Neill (2018), we presented a set of questions about population and climate change capturing elements of their statements. Table 4 gives an overview of this list of Likert-type items and these are ranked by the percentage of respondents who fully agree with each statement. The answers to statements 1 to 3 show that a large majority of respondents (91%) see climate change as the result of human action/behaviour; 85% believe saving the environment should have top priority even if this policy objective slows down economic growth; and 74% expect that climate change will lead to unprecedented migration flows. The consensus on this last issue is quite strong, and is in line with insights generated by research on climate migration (Hugo 2011; Kniveton et al. 2012; McLeman 2018; Robinson et al. 2020).

**Table 4:** Opinions of demographers on population and climate change, ranked by level of (full) agreement, 2020

Statements:	Fully disagree	Disagree	Neither agree, nor disagree	Agree	Fully agree	Don’t know	Total
	Percentages						
1.Climate change primarily result of human action	2	1	5	32	59	2	100
2. Saving the environment should be top priority at all costs	2	1	11	33	52	1	100
3. Climate change leads to unprecedented migration flows	1	9	11	49	25	6	100
4. Reducing global population is crucial to reduce CO2 emissions	9	25	28	26	10	3	100
5. Current world population size exceeds its carrying capacity	12	33	19	17	14	5	100
6. Family planning is by and large effective	5	25	26	26	5	15	100

*N* = 200. The questions on which respondents had to reflect were stated in full: (1) ‘Climate change is primarily the result of human action’; (2) ‘Saving the environment should be top priority for governments, even if this goal negatively affects economic growth’; (3) ‘Climate change will generate unprecedented migration flows across the globe’; (4) ‘Reducing the global population is a crucial in step in reducing global emissions of CO2’; (5) ‘The current size of the world population exceeds the carrying capacity of the earth’; (6) ‘Family planning policies to curb rapid population growth in developing countries are by and large effective’. Due to rounding errors, the sum of percentages may not always add up to 100

However, when we look at the connection between population and climate change, the consensus turns into a dissensus. The lack of consensus on the key role of population in climate change can be deduced from responses to the statement ‘Reducing the global population is a crucial step in reducing global emissions of CO_2_’: 36% of the respondents agree with this statement, and 34% disagree. In other words, demographers are thoroughly divided on this issue. The relationship between population and climate change is apparently not perceived by the respondents to be such a firm relationship as Bongaarts and O’Neill view it.

The responses to the statement ‘The current size of the world population exceeds the carrying capacity of the earth’ may explain the divided stance. 45% of the respondents disagree with this statement, and only 31% agree; in other words, close to half of our group of demographers is not convinced that the global population size matters. Apparently this is a rather stable evaluation, because the identical statement was used in a survey by Van Dalen and Henkens (2012), carried out in 2009 to a more or less similar result: 49% disagreed, and 33% agreed. Apparently quite a number of demographers think that population does not have much effect on climate. They may have adopted the optimistic view of a demographer like David Lam (Lam, 2011, 2013) or a non-demographer like the late statistician Hans Rosling that population growth is simply no longer a problem—or perhaps they believe that we have solved the population problem with human ingenuity. The concept of Earth’s carrying capacity is one that leaves much room for diverse interpretations, as it depends on technology, preferences and the structure of production and consumption (Arrow et al. 1995; Cohen 1997), but it nonetheless gives us an idea—albeit a very subjective one—whether the current global population size is sustainable.

Finally, turning to the effectiveness of family planning, one can again see how divided demographers can be. The statement ‘Family planning policies to curb rapid population growth in developing countries are by and large effective’ generates a high level of dissensus: 30% disagrees and 31% agrees, and the remaining 39% neither agrees nor disagrees. Of course, one could claim that judging the effectiveness of family planning requires some specialized knowledge. And indeed, when we divide the sample into demographers who have a high level of (self-reported) knowledge on fertility and family planning and we leave out those respondents who report not having an opinion (‘don’t know’), then the group of specialists scores significantly higher (42% thinks family planning is effective and 31% disagrees) than the group possessing low or medium-level knowledge in this domain (29% thinks family planning is effective and 40% disagrees). However, one could still argue that even among self-reported fertility experts the level of consensus is low. In short, one of the misperceptions that Bongaarts and O’Neill claims as being prevalent among the climate change community is also present among demographers.

Although these opinions are presented as being independent of each other, they are to a large degree correlated (see Table 5): views on the carrying capacity are closely related with opinions on taking the reduction of global population seriously. Likewise, the conviction that climate change is primarily the result of human action/behaviour is closely related to the opinion on the urgency of saving the environment at all costs. However, the low correlations between the items are equally noteworthy, especially the item concerning the statement that climate change should have top priority is very loosely correlated with the item expressing the view that the global population should be reduced at any cost (ρ = 0.20).

**Table 5:** Correlation matrix with climate change worries (from Table 1) and opinions on climate change causes and consequences (as summed up in Table 4)

	Worry climate change	Carrying capacity stress	Family planning effectiveness	Reduce global population	Climate change result of humans	Climate migration	Climate top priority
Worries climate change	1.00						
Carrying capacity stress	0.20***	1.00					
Family planning effectiveness	0.06	0.10	1.00				
Reduce global population	0.22***	0.61***	0.12	1.00			
Climate change result of human action	0.42***	0.15**	0.06	0.16**	1.00		
Climate leads to migration	0.31***	0.13*	0.01	0.16**	0.32***	1.00	
Climate top priority policy	0.51***	0.18**	0.05	0.20***	0.54***	0.34***	1.00

A final observation on Table 5 concerns the fact that the item measuring the effectiveness of family planning to curb rapid population growth in developing countries is uncorrelated with all the other items in the table. In other words, the perceived effectiveness of family planning programmes is not associated with a high sense of urgency and worry about climate change. This could be a tell-tale sign that demographers are not inclined to think about direct population policies—like family planning—in tackling climate change, and perhaps they are more focused on indirect policy measures, like stressing the importance of women’s agency. In Appendix Table 7, we analysed the link between characteristics of respondents and their views on the statements of Table 4. These supplementary analyses provide few clues as to why opinions on efforts on reducing the global population and family planning (statements 4 and 6 respectively) are so divided.

## Conclusion and Discussion

Demographers display a clear consensus on the importance of climate change and the urgency to act. However, a clear dissensus is visible when demographers focus on the potential of demographic developments and policies to address the challenges of climate change. Even though demographers perceive climate change primarily as the result of human action, this dissensus could be a troublesome divide, insofar as it signals that when it comes to taking action they do not see population policy as an effective policy instrument. ‘Population is left out in the cold’, as Bongaarts and O'Neill (2018) phrased it in their criticism of the assessments of the IPCC. We can only speculate about the reasons why so many demographers are reluctant when it comes to supporting population policies in averting the negative consequences of climate change. Three reasons spring to mind.

First, one cannot rule out the possibility that many demographers are just as uninformed as laymen when it comes to the issue of climate change. And judging from the research practice of demographers, this seems plausible. In most of the academic demography journals, the topics of ‘climate change’ or ‘global warming’ rarely make an appearance: only 1.4% of the articles that appeared in general oriented demography journals over the period 1990–2020 covered these topics.^6^ Given the fact that climate change is a relatively new research field in demography, it is to be expected that knowledge claims are quite uncertain because the research at the frontier of a field generates far less consensus than older fields that deal with settled issues.

A second reason for the dissensus among demographers, as put forward by Bongaarts and O’Neill (2018), is that family planning policies are too controversial to succeed. As mentioned earlier, they notice that ‘entering into a population policy discussion blames the poor countries for problems created by the rich countries’ (p. 652). In some ways, this argument is related to the arguments mentioned by Baer (2013) that efforts to reduce greenhouse gas emissions should not harm the poor countries’ ‘right to develop’. According to defenders of this right, individuals bear no moral responsibility to mitigate climate change. However, as Meyerson (1998) and Cafaro (2012) make clear, even such a right demands restrictive measures if it’s going to be universally applicable in future. In the current survey, we have respondents who are primarily European, and hence, in line with the arguments above, they may be more reluctant to emphasize the role of population growth in developing countries as one of the main drivers of CO_2_ emissions, either out of guilt or because they tacitly defend the right of developing countries to development.

A final reason why substantial dissensus exists between demographers about the use of family planning may be tied to the history of the discipline of demography. Despite all the efforts to focus in matters of population policies more on empowerment of women and their reproductive rights—as formally affirmed at the International Conference on Population and Development (ICPD) of 1994^7^—government intervention in matters of population is still associated by many with images of coercion and unethical practices by governments and NGOs (cf. Connelly (2008)). One need only think about the one-child policy of China as an example of state coercion in violating reproductive rights. The ethical side of demography tends to be forgotten, and perhaps this also explains why scholars ‘from the outside’ are less inhibited in making claims about population policy and seeing direct benefits of family planning, whereas demographers are still ambivalent. For outsiders, population policy may be perceived as a technical fix,^8^ whereas for academic demographers population policy is perhaps tainted by its neo-Malthusian roots. As Greenhalgh (1996) once formulated the central problem that demography has faced in its history of becoming a science: ‘demography both wants and needs to be an intellectual activity, remote from the problems of society; but it exists in an environment that constrains it to operate primarily as a policy-oriented field that is sometimes pushed into advocacy.’ (p. 32). In other (or our) words, demographers do not want to return to the role of policy advocate and would rather see population policy stimulate the use of the ‘force of reason’ (Sen, 1997) rather than the force of coercion.

The survey results we presented are a first step in looking at how demographers assess the population-climate change nexus. Needless to say, the survey does have a number of limitations. We did not assess opinions about the relationship about how climate change may have demographic consequences, in particular on fertility and mortality. Furthermore, this study has been carried out among European demographers, which limits the generalizability of their views to those of demographers living in other regions.

Despite these limitations, the overarching message remains simple and important: demographers express a clear consensus on the importance of climate change. This concern also ranks high on the list of worries and priorities of the general public. For instance, as registered by the Eurobarometer (2019), climate change is a topic that captures the minds of people and the need to take action. However, when it comes to taking corrective action, the community of demographers displays a clear dissensus on the potential of population policy and this may be a worry in itself. Usually, a dissensus is part and parcel of working at the forefront of science. However, in terms of the issue of climate change, time is not on the scientist’s side. As the global population increases and the climate and its effects on the environment seem to have taken an irreversible course, every year counts.

As Cohen (2010) once noted: ‘People are part of the problem of climate change and part of the solution’ (p. 158). The challenge for demographers in the coming years is to develop both more scientific consensus on the complex relationship between population and climate change as well as no-regret solutions that might persuade the skeptics of climate change or population policy.

## Appendix

**Table 6:** Descriptive statistics of a survey among members of EAPS

	Mean
Age groups	
25–34	19%
35–44	34%
45–54	17%
55–65	19%
65 +	11%
Gender (male = 0)	51%
Political orientation (× 10^–2^)	0.29 (s.d. 0.17)
Country groups	
Northern Europe	61%
Southern Europe	22%
Eastern Europe	10%
Outside Europe	7%

*N* = 186

**Table 7:** Explaining opinions of demographers on population and climate change

	Country of residence (North = 0)			Political orientation (× 10^–2^)	Gender (male = 0)	Pseudo-R^2^
	Southern Europe	Eastern Europe	Outside Europe			
1.Climate change primarily result of human action	−0.76	−0.62	0.10	−2.22*	−0.12	0.08
	(0.41)	(0.57)	(0.75)	(1.17)	(0.34)	
2. Saving the environment should be top priority at all costs	−0.66*	−0.94*	−0.03	−2.38**	0.18	0.07
	(0.37)	(0.55)	(0.66)	(1.04)	(0.32)	
3. Climate change leads to unprecedented migration flows	−0.26	−0.18	0.24	−0.33	0.14	0.04
	(0.38)	(0.57)	(0.66)	(0.98)	(0.31)	
4. Current world population size exceeds its carrying capacity	−0.46	0.06	−0.54	0.38	−0.13	0.02
	(0.47)	(0.49)	(0.64)	(0.90)	(0.29)	
5. Reducing global population is crucial to reduce CO_2_ emissions	−0.33	−0.37	−0.15	0.64	−0.22	0.02
	(0.35)	(0.51)	(0.59)	(0.92)	(0.29)	
6. Family planning is effective	−0.25	−0.10	−0.25	0.78	−0.71**	0.05
	(0.38)	(0.53)	(0.62)	(1.00)	(0.32)	

(a)These ordered logit estimates have also been controlled for age and professional rank within university/institute of respondents. The ‘don’t know’-category has been left out of the analyses. ****p* < 0.01; ***p* < 0.05; and **p* < 0.10. N varies between 160 and 187

.

## Footnotes

1 This was not the first time, as Bongaarts et al. (1997) also made this plea about the neglect of an earlier report by IPCC. Apparently, the neglect of population policy is a structural element in IPCC circles.

2 Although the survey was carried out partially during the outbreak of the Covid-19 virus, 65 per cent of the responses were collected before mid-March when most countries started to institute measures to contain the spreading of the virus. The economic consequences were at that time still far from clear. The questions did not concern Covid-19 directly, and we believe that because our respondents are experts in their field, their answers will not have been affected much by the pandemic.

3 For example, age is missing for 50 per cent of the EAPS members, as EAPS did not inquire into the age of new members; likewise, gender is missing for 4 per cent of the members, and country of residence is missing in the records for 10 per cent of the members.

4 What makes the current figure more difficult to compare to other studies is that in this survey respondents had to place themselves on the left or the right by sliding a scale. Other studies often have explicit groups with a middle category, which might be attractive for those who have no clear orientation.

5 Tables with simulations of priority rankings can be acquired upon request from the authors.

6 Search on Web of Science covering the following journals: *Demography, Population Studies, Demographic Research, European Journal of Population, Population, Population and Environment, and Population Research and Policy Review*. Only 159 articles out of 11,229 articles covered the topics ‘climate change’ or ‘global warming’ in the period 1990–2020 (May).

7 The adopted Programme of Action at this conference stressed that all people should have access to comprehensive reproductive health care, including voluntary family planning. Reproductive health rights of women were seen as a precondition for empowering women and subsequent well-being.

8 Compare e.g. the views of ecologists like Hardin (1968) and Ehrlich (1968) or the more recent outsiders’ views as found e.g. in Ripple et al. (2017), all of which allude to some optimal population size.

## References

[CR1] Arrow K, Bolin B, Costanza R, Dasgupta P, Folke C, Holling CS (1995). Economic growth, carrying capacity, and the environment. Ecological economics.

[CR2] Baer P (2013). The greenhouse development rights framework for global burden sharing: Reflection on principles and prospects. Wiley Interdisciplinary Reviews: Climate Change.

[CR3] Bongaarts, J. (1992). Population growth and global warming. *Population and Development Review*, *18*(2), 299–319.

[CR4] Bongaarts J, O'Neill BC (2018). Global warming policy: Is population left out in the cold?. Science.

[CR5] Bongaarts J, O'Neill BC, Gaffin SR (1997). Global warming policy: Population left out in the cold. Environment.

[CR6] Cafaro P (2012). Climate ethics and population policy. Wiley Interdisciplinary Reviews: Climate Change.

[CR7] Carlton JS, Perry-Hill R, Huber M, Prokopy LS (2015). The climate change consensus extends beyond climate scientists. Environmental Research Letters.

[CR8] Cohen JE (1997). Population, economics, environment and culture: an introduction to human carrying capacity. Journal of Applied Ecology.

[CR9] Cohen JE (2010). Population and climate change. Proceedings of the American Philosophical Society.

[CR10] Connelly, M. J. (2008). *Fatal misconception: The struggle to control world population*. Harvard University Press.

[CR11] Crist E, Mora C, Engelman R (2017). The interaction of human population, food production, and biodiversity protection. Science.

[CR12] Dyson T (2005). On development, demography and climate change: The end of the world as we know it?. Population and environment.

[CR13] Ehrlich, P. R. (1968). *The population bomb*. Ballantine.

[CR14] Eurobarometer (2019). Special Eurobarometer: Climate Change. *Wave EB91.3* Brussels: European Commission.

[CR15] Gerlagh, R., Lupi, V., & Galeotti, M. (2018). Family planning and climate change. *CESifo working papers*. Munich.

[CR16] Greenhalgh S (1996). The social construction of population science: An intellectual, institutional, and political history of twentieth-century demography. Comparative studies in society and history.

[CR17] Gross N, Fosse E (2012). Why are professors liberal?. Theory and Society.

[CR18] Guillebaud, J. (2016). Voluntary family planning to minimise and mitigate climate change. *bmj, 353*, i2102. 10.1136/bmj.i2102 27207903

[CR19] Hardin G (1968). The tragedy of the commons. Science.

[CR20] Hugo G (2011). Future demographic change and its interactions with migration and climate change. Global Environmental Change.

[CR21] Klein DB, Stern C (2005). Professors and their politics: The policy views of social scientists. Critical Review.

[CR22] Kniveton DR, Smith CD, Black R (2012). Emerging migration flows in a changing climate in dryland Africa. Nature Climate Change.

[CR23] Lam D (2011). How the world survived the population bomb: Lessons from 50 years of extraordinary demographic history. Demography.

[CR24] Lam D (2013). Reply to Stan Becker, “has the world really survived the population bomb?(Commentary on “how the world survived the population bomb: lessons from 50 years of extraordinary demographic history”)”. Demography.

[CR25] Lutz W (2017). How population growth relates to climate change. Proceedings of the National Academy of Sciences.

[CR26] May AM, McGarvey MG, Kucera D (2018). Gender and European Economic Policy: A Survey of the views of European Economists on Contemporary Economic Policy. Kyklos.

[CR27] McLeman R (2018). Thresholds in climate migration. Population and environment.

[CR28] Meyerson FA (1998). Population, development and global warming: Averting the tragedy of the climate commons. Population and environment.

[CR29] O'Neill BC, Liddle B, Jiang L, Smith KR, Pachauri S, Dalton M (2012). Demographic change and carbon dioxide emissions. The Lancet.

[CR30] O'Neill, B. C., MacKellar, F. L., & Lutz, W. (2005). *Population and climate change*: Cambridge University Press.

[CR31] Ripple WJ, Wolf C, Newsome TM, Galetti M, Alamgir M, Crist E (2017). World scientists’ warning to humanity: A second notice. BioScience.

[CR32] Robinson C, Dilkina B, Moreno-Cruz J (2020). Modeling migration patterns in the USA under sea level rise. PLoS ONE.

[CR33] Sen A (1997). Population policy: Authoritarianism versus cooperation. Journal of Population Economics.

[CR34] Van Dalen HP (2019). Values of economists matter in the art and science of economics. Kyklos.

[CR35] Van Dalen HP, Henkens K (2012). What is on a demographer’s mind? A worldwide survey. Demographic Research.

[CR36] Van Dalen, H. P., & Henkens, K. (2020). When is fertility too low or too high? Population policy preferences of demographers around the world. *Population Studies*. 10.1080/00324728.2020.1784986. 10.1080/00324728.2020.1784986 32697143

[CR37] Van de Werfhorst HG (2020). Are universities left-wing bastions? The political orientation of professors, professionals, and managers in Europe. The British Journal of Sociology.

